# Perceptions of and support for national health insurance in South Africa’s public and private healthcare sectors

**DOI:** 10.11604/pamj.2018.30.277.14147

**Published:** 2018-08-16

**Authors:** Frederik Booysen, Charles Hongoro

**Affiliations:** 1 Population Health, Health Systems and Innovation (PHHSI) Research Programme, Human Sciences Research Council (HSRC), Pretoria, South Africa; 2 Department of Economics, University of the Free State (UFS), Bloemfontein, South Africa; 3 Faculty of Health Sciences, Fort Hare University, East London, South Africa

**Keywords:** Healthcare, medical aid, national health insurance, South Africa

## Abstract

**Introduction:**

For the purpose of effective implementation of a National Health Insurance (NHI) policy it is necessary to have an understanding of the awareness and perceptions of and support for such policy among clients using the healthcare system.

**Methods:**

The South African National Health and Nutrition Examination Survey asked household heads a series of questions on healthcare utilisation and access and collected information on knowledge and perceptions of and support for national health insurance. Comparisons are drawn between private sector healthcare users with medical aid and public sector healthcare users without medical aid, using descriptive and regression analysis.

**Results:**

Inequalities in access to quality healthcare remain stark. Only 8.5% of private users had postponed seeking healthcare compared to 23.9% of public users (p < 0.001). Only 11.9% of public users were very satisfied with the quality of healthcare services compared to 50.2% of private users (p < 0.001). More than eighty percent of healthcare users however were of the opinion that NHI is a top priority. However, for healthcare users to sacrifice choice required a national health insurance that provides better quality healthcare, increasing the probability of support for an NHI with lower cost and full coverage by 10.1%.

**Conclusion:**

It is imperative to provide better quality healthcare services in the public sector for private sector users to be supportive of national health insurance. Concerted efforts are also required to develop a proper communication strategy to disseminate information on and garner support for national health insurance, both in the public and private healthcare sectors.

## Introduction

Inequalities and inequity in South Africa between the public and private healthcare sectors in terms of availability, acceptability and affordability is well documented [1, 2]. In response to the quest for universal health coverage, government published the Green Paper on National Health Insurance (NHI) in August 2011. In December 2015, government launched the White Paper, which was officially adopted in June of 2017, thus heralding the start of the long awaited implementation of NHI. In the process of implementing such a policy, or any public policy for that matter, it is important to have a grasp of healthcare users' awareness and knowledge and perceptions of and support for NHI. Only a handful of studies have documented the awareness and/or perceptions of and support for national health insurance among healthcare users in South Africa. An early nationally representative survey fielded in 2005 documented a majority but not universal support for national health insurance [3]. In 2008, in the early run-up to the publication of the Green Paper [4], a nationally representative survey found that users in both the public and private sectors are dissatisfied with healthcare services, which in the authors' opinion signifies that South Africans are ready for a health systems change of this nature. A more recent three-province survey conducted in 2013 shows that awareness of the NHI was generally good, expectations high, but knowledge poor [5-7]. This paper sets out to in more detail explore the significant differences in awareness, knowledge and perceptions of and support for national health insurance in South Africa among healthcare users in the private and public sectors, using data from a large nationally representative survey. In addition, the paper explores how access, awareness and perceptions regarding NHI are associated with support for such policy.

## Methods

The South African National Health and Nutrition Examination Survey (SANHANES) is a nationally representative, cross-sectional survey with a multi-stage disproportionate, stratified cluster sampling design. Among the 8,168 valid, occupied households at the 10,000 sampled visiting points from 500 Enumerator Areas (EAs), a total of 6,306 (77.2%) were interviewed [8]. The survey was conducted in 2012 and the Human Sciences Research Council's (HSRC) ethics committee approved the study. Written informed consent was obtained from all study participants. This study was an analysis of secondary data and did not require any ethical approval. In SANHANES's household questionnaire, answered by the household head, a variety of questions on health care utilisation and access was followed by a question asking respondents, “In the past 6 months, have you seen, read or heard any news or information about a proposal by government to introduce a programme to provide national health insurance for all South Africans.” Following on this question respondents were provided with the following statement: “We are now going to talk about some of the changes government is planning with regard to health care in South Africa. The government wants to create a National Health Insurance, which is a system in which everyone is covered by health insurance and people contribute according to ability to pay and use health services according to their need.” Subsequently, respondents were asked a total of eleven questions on various aspects regarding national health insurance and healthcare financing. The main emphasis of this paper is on the comparative views of two important groups of constituents, namely public sector users without medical aid (n = 3,912) versus private sector users with medical aid (n = 1,156), giving a total sample size of 5,068. The statistical analysis comprises two components. First, the following four sets of outcomes are compared across the two groups of healthcare users: (i) access to and satisfaction with healthcare; (ii) awareness and knowledge of national health insurance; (iii) perceptions of national health insurance; and (iv) support for national health insurance, using X^2^ and t-tests. In all cases, don't know responses are treated as missing. Secondly, a series of probit regression models are estimated to determine the extent to which various opinions of national health insurance predicts support for the policy when adjusting for access and awareness. The regression models take the following form: Pr (Y = 1|X) = Φ (X^T^β), where Pr is a probability, Y is a dichotomous outcome coded as yes (= 1) or no (= 0), i.e. Yi {0, 1}; Φ is the cumulative distribution function (CDF) of the standard normal distribution; and X^T^ a vector of independent explanatory variables. More specifically, these variables include three sets of binary categorical variables: (i) healthcare user type (private sector user = 1, public sector user = 0); (ii) have information regarding NHI (yes = 1, no = 0); and (iii) perceptions of NHI regarding five matters, i.e. affordability, cost, financial benefits, benefits to the country and quality of healthcare services (yes=1, no = 0). Analysis was conducted using Stata13 software. The minimum criterion for statistical significance is the 95% level (i.e. p < 0.05).

## Results

There were stark and statistically significant differences between healthcare users in terms of access to healthcare (Table 1). Compared to private sector users with medical aid, almost three times as many public sector users without medical aid reported having to postpone receiving healthcare in the past year (8.5% versus 23.9%, p < 0.001), while more than twice as many reported experiencing difficulties with affording the cost of healthcare (14.1% versus 30.9%, p < 0.001) or prescription medicine (13.0% versus 29.6%, p < 0.001) in the past 12 months. Eleven percent more private sector users with medical aid lived within close reach (0-10km) of a healthcare facility (76.0% versus 85.0%, p < 0.001). In turn, a larger proportion of public sector users without medical aid lived more than 10km away from a healthcare facility (24.0% versus 15.0%, p < 0.001). Table 2equally reveals vast differences in satisfaction with healthcare. Many more private sector users with medical aid were “very satisfied” with the quality and cost of their healthcare. The quality gap was particularly large: approximately half of private sector users with medical aid (50.2%) was “very satisfied” compared to only 11.9% of public sector users without medical aid. Concomitantly, more public sector users without medical aid were only “satisfied” rather than “very satisfied” with the quality and cost of healthcare (49.1% versus 44.3%). Another indication of the quality divide between the public and private sectors is that many more respondents without medical aid were indifferent about the quality of care they receive in the public sector (17.1% versus 3.1%), or “dissatisfied” or “very dissatisfied” (21.9% versus 2.5%) when compared to private sectors users with medical aid (p < 0.001). The perceived quality divide between the private and public healthcare sectors therefore remained stark in the eyes of healthcare users. The differences for cost of healthcare were less pronounced but remained highly statistically significant (p < 0.001). The results were as follows: very satisfied (29.7% versus 13.0%); satisfied (39.9% versus 48.5%); neither satisfied nor dissatisfied (12.4% versus 21.6%); dissatisfied (12.3% versus 11.9%); very dissatisfied (5.8% versus 5.0%). Only approximately one in five respondents (20.4%) had knowledge of or information on the national health insurance policy (Table 3). There was a huge divide moreover between the two groups of public (13.3%) and private sector users (44.7%) in terms of awareness of the national health insurance policy (p < 0.001). Among those public sector users without medical aid who were aware of the policy, a somewhat greater number reported having either “a little” or “not yet enough” information when compared to private sector users with medical aid (56.3% versus 49.3%), among whom a larger proportion had “a fair amount” of knowledge (34.3% versus 26.4%). These differences however were not statistically significant (p = 0.078).

**Table 1:** Healthcare access (%)

	Public sector users with no medical aid	Private sector users with medical aid	Total
Postponed care	23.9	8.5	21.0
Difficulty affording cost of healthcare	30.9	14.1	27.2
Difficulty affording prescription medicine	29.6	13.0	25.7
Distance to nearest healthcare facility:			
0-10 kilometres	76.0	85.0	77.7
11-20 kilometres	18.1	11.0	16.3
21-30 kilometres	3.9	2.5	3.6
>30 kilometres	2.1	1.5	2.1
Total	100.0	100.0	100.0

Note: results are weighted. ‘Total’ figure represents all respondents in the full sample.

Statistical significance for all comparisons: p<0.001

**Table 2:** Satisfaction with healthcare (%)

	Public sector users with no medical aid	Private sector users with medical aid	Total
Satisfaction with quality of healthcare:			
Very satisfied	11.9	50.2	21.2
Satisfied	49.1	44.3	48.4
Neither satisfied nor dissatisfied	17.1	3.0	13.6
Dissatisfied	14.6	1.8	11.4
Very dissatisfied	7.3	0.7	5.5
Total	100.0	100.0	100.0
Satisfaction with cost of healthcare:			
Very satisfied	13.0	29.7	16.5
Satisfied	48.5	39.9	46.7
Neither satisfied nor dissatisfied	21.6	12.4	19.1
Dissatisfied	11.9	12.3	12.3
Very dissatisfied	5.0	5.8	5.4
Total	100.0	100.0	100.0

Note: results are weighted. ‘Total’ figure represents all respondents in the full sample.

Statistical significance for all comparisons: p<0.001

**Table 3:** Awareness and knowledge of national health insurance (%)

	Public sector users with no medical aid	Private sector users with medical aid	Total	p-value
Have information on NHI	13.3	44.7	20.4	<0.001
**Level of knowledge**				
A lot	17.8	16.3	17.6	0.078
A fair amount	26.6	34.2	31.4	
A little	35.6	32.6	33.6	
Not yet enough	20.0	16.9	17.4	
Total	100.0	100.0	100.0	

Note: level of knowledge is reported only for those with information on the NHI

Results are weighted. ‘Total’ figure represents all respondents in the full sample

Perceptions on national health insurance (Table 4) differed statistically significantly between the two groups of healthcare users in almost all respects (p < 0.001). The exception was views on the affordability of the policy, where users were in agreement. Almost three-quarters of users was of the opinion that national health insurance is affordable (74.3% versus 74.7%, p = 0.357). Public sector users without medical aid were significantly more likely to trust government to run the new health insurance scheme in comparison to private sector users with medical aid, of whom nearly half put their trust in a private organisation (79.2% versus 48.9%, p < 0.001). In the case of the other issues, private sector users with medical aid had less positive views of national health insurance than public sector users without medical aid, i.e. fewer felt that national health insurance would be cheaper (62.1% versus 75.8%, p < 0.001), that their family would be better off (63.3% versus 75.2%, p<0.001), that the country would be better off (70.4% versus 76.6%, p < 0.001), or that the quality of healthcare would improve under national health insurance (62.3% versus 79.6%, p < 0.001). The difference in opinion was most pronounced for perceptions of the quality of healthcare to be provided under the NHI, i.e. 17.3 percentage points. The reported differences in support for national health insurance mirrored the above differences in perceptions, i.e. there was less support for the new policy among private sector users with medical aid than among public sector users without medical aid (Table 4). These differences were statistically significant in all but one instance. Fewer private sector users with medical aid were of the opinion that NHI is a top priority and that insurance for all is the priority (as opposed to making healthcare better and more affordable) (79.1% versus 86.3%, p = 0.001). In addition, relatively fewer supported a national health insurance that lowered healthcare costs and provided coverage to all South Africans, but limited the choice of doctor, hospital, or treatment (60.4% versus 75.8%, p < 0.001). Relatively fewer private sector users with medical aid preferred national health insurance over the current medical aid system (61.1% versus 73.1%, p < 0.001). The one exception, however, in terms of the direction of the relationship and its statistical significance, was the question of whether health insurance for all remains important even if taxes increase. In fact, a greater though not significantly higher percentage of private sector users with medical aid were in support of this statement compared to public sector users without medical aid (64.4% versus 61.0%, p = 0.072). Here, the greatest divide in perceptions (i.e. 15.4 percentage points), pertained to the question of cost and coverage versus choice of healthcare services. The regression results are presented in Table 5.

**Table 4:** Perceptions of and support for national health insurance (%)

	Public sector users with no medical aid	Private sector users with medical aid	Total	p-value
A. Perceptions				
Government should implement NHI	79.2	48.9	72.1	<0.001
NHI is affordable	74.3	74.7	73.9	0.357
NHI is cheaper than current arrangement	75.8	62.1	73.0	<0.001
Family financially better off under NHI	75.2	63.3	72.1	<0.001
Country better off under NHI	76.6	70.4	75.0	<0.001
Better quality of care under NHI	79.6	62.3	75.0	<0.001
B. Support				
NHI is a top priority	86.3	79.1	84.3	0.001
Insurance for all is the priority	53.3	40.5	49.6	<0.001
NHI is important even if taxes increase	61.0	64.4	61.3	0.072
Prefer NHI over current medical aid system	73.1	61.1	70.2	<0.001
Support NHI with lower cost but less choice	75.8	60.4	71.3	<0.001

Note: percentages are share of respondents who responded in the affirmative to the relevant question. Results are weighted. ‘Total’ figure represents all respondents in the full sample

**Table 5:** Predictors of support for national health insurance

	Dependent variables (yes/no):				
Independent variables	a. NHI is a top priority	b. Insurance for everyone is the priority	c. NHI is important even if taxes increase	d. Prefer NHI over current medical aid system	e. Prefer NHI with lower cost and full coverage, but less choice
Private sector user with medical aid	-0.016 (0.025)	-0.089 (0.037) *	0.038 (0.038)	-0.114 (0.034) ***	-0.055 (0.031)
Aware of NHI	-0.026 (0.021)	-0.062 (0.031)	0.000 (0.033)	-0.017 (0.030)	-0.108 (0.027) ***
NHI is affordable	0.150 (0.020) ***	0.006 (0.033)	0.134 (0.033) ***	0.042 (0.031)	0.077 (0.028) **
NHI is cheaper than current medical aid system	0.056 (0.019) **	0.022 (0.032)	0.036 (0.033)	0.081 (0.032) *	0.108 (0.028) ***
Family is financially better off under NHI	0.036 (0.025)	0.015 (0.045)	0.071 (0.045)	0.005 (0.044)	0.137 (0.038) ***
Country is better off under NHI	0.061 (0.026) *	-0.029 (0.046)	0.054 (0.047)	0.074 (0.045)	-0.009 (0.044)
Quality of healthcare is better under NHI	0.020 (0.025)	0.047 (0.041)	0.080 (0.041)	0.014 (0.040)	0.101 (0.035) **
Wald Χ^2^ statistic (p-value)	192.68 (<0.001)	25.79 (0.018)	93.72 (<0.001)	62.38 (<0.001)	150.86 (<0.001)
Pseudo R^2^	0.140	0.012	0.044	0.034	0.106
Correct predictions (%)	85.8	55.0	66.1	70.9	78.1
Sample (n)	3,050	3,055	2,847	2,842	3,011

Notes: results are weighted. The independent variables are all binary in nature (yes/no). The comparison group for ‘private sector user with medical aid’ is ‘public sector user with no medical aid’, ‘make healthcare better and more affordable’ for ‘insurance for everyone is the priority’ and ‘no’ for all other outcomes. Adjusted for household head’s age, sex and race. Results are for probit regression models and are reported as marginal effects calculated at the mean. Robust standard errors are reported in parentheses. Statistical significance:

* p<0.05

** p<0.01

*** p<0.001

The regression models performed adequately in terms of overall fit, i.e. the p-values for the Wald X^2^ statistic were all below 0.05 and generally smaller than 0.001. The models' predictive power however was low but acceptable in terms of the percentage of positive outcomes predicted correctly by the specific regression model. According to the regression results, public sector users without medical aid expressed a significantly stronger choice in terms of preferring the proposed national health insurance over the current medical aid system (d) or considering NHI for everyone as a top priority (b). Being a private sector user with medical aid decreased the probability of a preference for NHI over the current medical aid system by a significant 11.4% and by 8.9% for considering insurance for everyone being the top priority. In other words, private users with medical aid were more in favour of the current medical aid system than a new national health insurance. The same was true for insurance for everyone being the top priority. Awareness predicted only one outcome, namely the preference for cost and coverage over choice. More specifically, those who at the time were aware of the national health insurance policy were 10.8% less probable to accept a national health insurance option that is less costly and ensures full coverage, but limits choice of healthcare provider or treatment. This particular result is plausible insofar as private sector users have been shown to be less supportive of NHI (Table 4), but more likely to have information on the NHI (Table 3). The single most important predictors of support for NHI in terms of perceptions were views regarding its cost and affordability. Being of the opinion that NHI is affordable significantly increased the probability of being of the opinion that NHI is a top priority (a) and that NHI is important even if taxes increase (c), by 15.0% and 13.4%, respectively, and preferring NHI with lower cost and full coverage, but less choice (e), by 7.7%. Likewise, being of the opinion that the NHI is cheaper than the current medical aid system increased not only the probability of being of the opinion that NHI is a top priority (a) by 5.6%, and that a NHI with lower cost and full coverage, but less choice is preferred (e), by 10.8%, but so too the probability of preferring the proposed NHI over the current medical aid system (d), by 8.1%. The probability of support for NHI as top priority was also enhanced when the NHI was perceived to make the country better off, in this case by 6.1%. The probability of support for an NHI that is less costly and ensures full coverage, but offers less choice (e), was also influenced by two other factors, namely whether such policy was perceived to have very direct benefits, i.e. making one's family better off financially, and whether the care provided under NHI was of a better quality. In these two instances, the probability of support increased by 13.7% and 10.1%, respectively.

## Discussion

This study draws comparisons between public sector healthcare users with no health insurance and private sector healthcare users with health insurance. These comparisons are important for the following reasons: the former group represents the main target beneficiaries of the new policy. The perceptions of the latter group is also particularly important in the context of NHI insofar as this group is impacted substantially by the proposed policy changes. These healthcare users, in accordance with the policy, have to switch from private medical aid schemes to national health insurance to receive the service benefits covered under the policy and possibly also to use both private and public healthcare services. These healthcare users may also be affected adversely by the tax implications of financing the NHI and in addition may continue to incur medical insurance costs where they choose to take out complementary insurance covering other excluded benefits. There are four principal findings. In the first instance, this study illustrates the entrenched inequalities in the South African healthcare system that serves to substantiate calls for and the necessity of policy initiatives such as national health insurance to achieve universal health coverage. The second finding to highlight is that awareness of the NHI at the time was considerably low, especially among the policy's main intended beneficiaries, namely public sector healthcare users with no medical aid. Yet, even less than half of private sector users was not aware of the policy, while of these, half or more described their knowledge as “a little” or “not yet enough”. These low levels of awareness and knowledge may be attributed to the fact that this survey was conducted at the very outset of the launch of the new policy, when one would not expect awareness to be very high. Nevertheless, others have also documented low levels of knowledge, despite reporting high levels of awareness [5-7]. A third main finding is that support for NHI, not surprisingly, is greater among those perceived to gain the most from the policy (improved access and financial protection) as opposed to those perceived to potentially stand to lose (higher taxes and less choice with perceived fewer benefits). In most respects, therefore, private sector users with medical aid are more pessimistic and less supportive of national health insurance than are public sector users with no medical aid.

The two issues on which the two groups of healthcare users are most divided are the issues of quality and choice. Nevertheless, a sizeable proportion of private sector users, one may say a majority, are positive and supportive towards the NHI. The research, in the final instance, also reveals how perceptions regarding the NHI's cost and affordability and its direct benefits and impact on the quality of healthcare services drives perceived support for the new policy. For healthcare users to sacrifice choice requires a national health insurance that is affordable, cheaper than the current medical aid system, makes families financially better off and provides quality healthcare services, increasing the probability of support for an NHI with lower cost and full coverage. Priority should therefore be given to ensuring the provision of quality healthcare services in the public sector. This is in line with what other authors have concluded, namely that, “public support for pre-payment is unlikely to be forthcoming unless there is confidence in the availability of quality health services” [4]. An important strength of this study is that SANHANES collected much more detailed information on awareness, knowledge, perceptions and support regarding national health insurance than did other surveys reported in the literature [3-7]. In addition, none of the studies published to date [3-7] provides a comparison of the survey responses of these two important groups of constituents, i.e. private sector users with medical aid and public sector users without medical aid, nor do these studies explore the associations between access, awareness, perceptions and support for national health insurance. In this way, this study complements research on this topic conducted to date. An important limitation however has to be kept in mind when interrogating these results. The response rate to the household survey was relative low (77.2%). When excluding “don't know” answers to the questions on national health insurance from the analysis, non-response increases further, primarily one may assume due to the reported limited awareness and knowledge of NHI on the part of respondents. Imperative, at this early stage of implementation, is to conduct an expanded survey(s) to continuously gauge support, knowledge, awareness, perceptions, behaviour and satisfaction with national health insurance, building on other studies [9-11], including replicating the NHI survey module in SANHANES. It furthermore is critical to, through further research, investigate how these dynamics in awareness, knowledge, perceptions and support translate into specific healthcare seeking behaviours or changes therein, particularly at the interface of the private and public sectors. Discrete choice experiments (DCEs) are particularly important in this regard as are surveys, which respectively elucidate stated and revealed preferences for healthcare. Prospective studies are required to monitor the impact on access and inequalities and quality of healthcare of the NHI's implementation.

## Conclusion

Concerted efforts are required to develop a proper communication strategy to disseminate information on the country's national health insurance policy and its implementation to healthcare users, both in the private sector and in the public sectors. In particular, a tailor-made communications strategy is required in the private sector to address the reported resistance to the proposed policy changes. What is paramount, moreover, is that evidence on the benefits and success of the NHI policy be interrogated by researchers and be made available in the public domain for stakeholders and citizens to draw informed conclusions regarding their support for this policy as implementation proceeds. This is so insofar as support for national health insurance in South Africa hinges on its cost and affordability, its direct financial benefit to families, and the quality of healthcare it provides to clients.

### What is known about this topic

- Awareness of national health insurance is high but knowledge poor;
- People generally are supportive of initiatives such as national health insurance.

### What this study adds

- Both awareness and knowledge of national health insurance is low;
- Private sector users with medical aid have poorer perceptions of and exhibit less support for national health insurance than public sector users without medical aid;
- Perceptions of affordability, financial benefit and quality of healthcare are important drivers of support for national health insurance

## Competing interests

The authors declare no competing interests.
